# Prophylactic anticoagulant therapy is associated with improved survival in ICU patients with non-COVID-19 pneumonia: a retrospective cohort study

**DOI:** 10.3389/fphar.2025.1597885

**Published:** 2025-05-08

**Authors:** Haiming Hu, Yixing Zhu, Ruikang Hu, Lijuan Zhao, Xiangwen Zeng, Xue Wu, Rui Qiu, Yihao Nie, Lokesh Sharma, De Chang

**Affiliations:** ^1^ Department of Pulmonary and Critical Care Medicine at The Seventh Medical Center, College of Pulmonary and Critical Care Medicine of The Eighth Medical Center, Chinese PLA General Hospital, Beijing, China; ^2^ Graduate School of Chinese PLA General Hospital, Beijing, China; ^3^ Senior Department of Oncology, The Fifth Medical Center of PLA General Hospital, Beijing, China; ^4^ Sanquan College, Xinxiang Medical College, Xinxiang, Henan, China; ^5^ Division of Pulmonary, Allergy, Critical Care, and Sleep Medicine, Department of Medicine, University of Pittsburgh School of Medicine, Pittsburgh, PA, United States

**Keywords:** non-COVID-19 pneumonia, prophylactic anticoagulant therapy, mortality, ICU, MIMIC database

## Abstract

**Background:**

Coagulation disorders are common complications in patients with pulmonary infections. Studies have suggested beneficial effects of anticoagulant therapies in patients with COVID-19. However, the usefulness of prophylactic anticoagulant therapies in patients with non-COVID-19 pulmonary infections is still a matter of debate. This study aimed to assessed the impact of prophylactic anticoagulant therapy in ICU patients with non-COVID-19 pneumonia.

**Methods:**

Patients were identified from the Medical Information Mart for Intensive Care-IV database. Propensity score matching (PSM) was utilized to minimize differences. Kaplan-Meier survival analysis was performed to assess mortality. Univariate and multivariate Cox regression models were used to identify prognostic factors for short-term mortality (7-day). The E-value was calculated to unmeasured confounding. To further explore the optimal anticoagulant administration, subgroup analyses were performed. We also explored the optimal administration strategies including the timing and duration of anticoagulant therapy.

**Results:**

A total of 1,000 ICU patients were included, with 500 receiving prophylactic anticoagulation therapy and 500 not. Both 7-day mortality (7.6% vs. 19.6%; p < 0.001) and 30-day mortality (19.6% vs. 31.2%; p < 0.001) in the anticoagulant group were lower than non-users. Kaplan-Meier survival analysis also showed a significantly lower prevalence of short-term mortality in patients who used anticoagulants. Both univariate (HR, 0.36; 95% CI, 0.25-0.53; p < 0.001) and multivariate (HR, 0.30; 95% CI, 0.21-0.44; p < 0.001) Cox regression analyses consistently demonstrated a significant reduction in short-term mortality associated with anticoagulation therapy. Subgroup analysis revealed that anticoagulant therapy was associated with reduced short-term mortality across most subgroups. Further analysis showed that late (≥6 h) and non-short-term (≥7 days) anticoagulation therapy were more effective.

**Conclusion:**

Our results support the potential value of prophylactic anticoagulation therapy as a strategy to improve survival in ICU patients with non-COVID-19 pneumonia.

## 1 Introduction

Pneumonia can be divided into two primary forms: community-acquired pneumonia (CAP) and hospital-acquired pneumonia (HAP), both of which are characterized by inflammatory responses and cell infiltration leading to alveolar injury (Torres et al., 2021). The clinical manifestations of pneumonia are diverse, spanning from mild symptoms such as cough and fever to severe complications like acute respiratory distress syndrome (ARDS) and even death (Vaughn et al., 2024). Approximately 489 million individuals were affected by lower respiratory tract infections in 2019, which resulted in an estimated 2.6 million deaths (GBD 2019 Diseases and Injuries Collaborators, 2020). Despite advancements in antimicrobial therapies and life-support measures for medicine, clinical outcomes have not significantly improved owing to aging demographics and the rise of drug-resistant pathogens (Torres et al., 2019).

Pneumonia treatment primarily encompasses pathogen-specific antibacterial or antiviral therapies (Reyes et al.,2023), as well as life-support interventions. Given that an exacerbated inflammatory response plays a key pathological role in severe pneumonia, glucocorticoids may be administered to mitigate inflammatory tissue injury (Pirracchio et al., 2024). However, given its immunosuppressive effects, the clinical utility of glucocorticoids still needs to be evaluated (Bergmann et al., 2023). Beyond the hyperinflammatory state, a hypercoagulable state is often associated with pneumonia-associated sepsis, as shown for COVID-19 by our group (Xiao et al., 2024). Numerous high-quality studies in COVID-19 patients have shown that prophylactic or therapeutic doses of anticoagulant therapy can effectively diminish the risk of thrombosis and markedly enhance survival rates (Lawler et al.,2021; Bradbury and McQuilten, 2022). Similar to COVID-19, critical illness and other infectious diseases often induce clotting disorders through the activation of pathogen-associated molecular patterns (PAMPs) and damage-associated molecular patterns (DAMPs) signaling (Yong and Toh, 2024). Blood coagulation and inflammation are universal responses to infection and there is crosstalk between inflammation and coagulation. Inflammatory cytokines and leukocyte elastase can downregulate natural anticoagulant proteins. These proteins are crucial for maintaining endothelial-cell integrity, regulating clotting processes, inhibiting vasoactive peptides, and attenuating leukocyte infiltration into the vessel wall (Esmon, 2004). Anticoagulant therapy was found that not only exerts an anticoagulant effect but also mitigates the inflammatory response by reducing the activation of NF-κB (Levy et al., 2016). Peptide fragments resulting from the proteolytic degradation of antithrombin AT exhibit antibacterial properties (Schlömmer et al., 2021). In theory, anticoagulant therapy can ameliorate multiple deleterious effects caused by infection-induced clotting, limiting the inflammatory sequelae in pneumonia and avoiding a vicious cycle of infection, coagulation and inflammation (Allingstrup et al., 2016). Unfortunately, the majority of research regarding the beneficial effects of anticoagulation therapy have been concentrated in animal models (Welty-Wolf et al., 2001; Barazzone et al., 1996). Meanwhile, population-based studies have primarily focused on COVID-19, with limited attention given to non-COVID-19 pneumonia.

In this retrospective cohort study, we assessed the impact of prophylactic anticoagulant therapy in ICU patients with non-COVID-19 pneumonia. Additionally, we explored the conditions under which anticoagulant may yield optimal clinical benefits.

## 2 Methods and materials

### 2.1 Data source

This retrospective study utilized patient data from the Medical Information Mart for Intensive Care Ⅳ (MIMIC-Ⅳ, version 3.0) (Goldberger et al., 2000; Johnson et al., 2023). MIMIC-IV is an extensive, publicly accessible database that contains comprehensive health records from 546,028 patients who were admitted to critical care units at Beth Israel Deaconess Medical Center (Johnson, 2024). Author Haiming Hu completed the necessary training and passed the examination mandated by the National Institutes of Health (NIH), and subsequently obtained access to the database to extract the necessary data (Record ID: 13485805). No additional ethical approval was required.

### 2.2 Criteria for population selection

**FIGURE 1:**
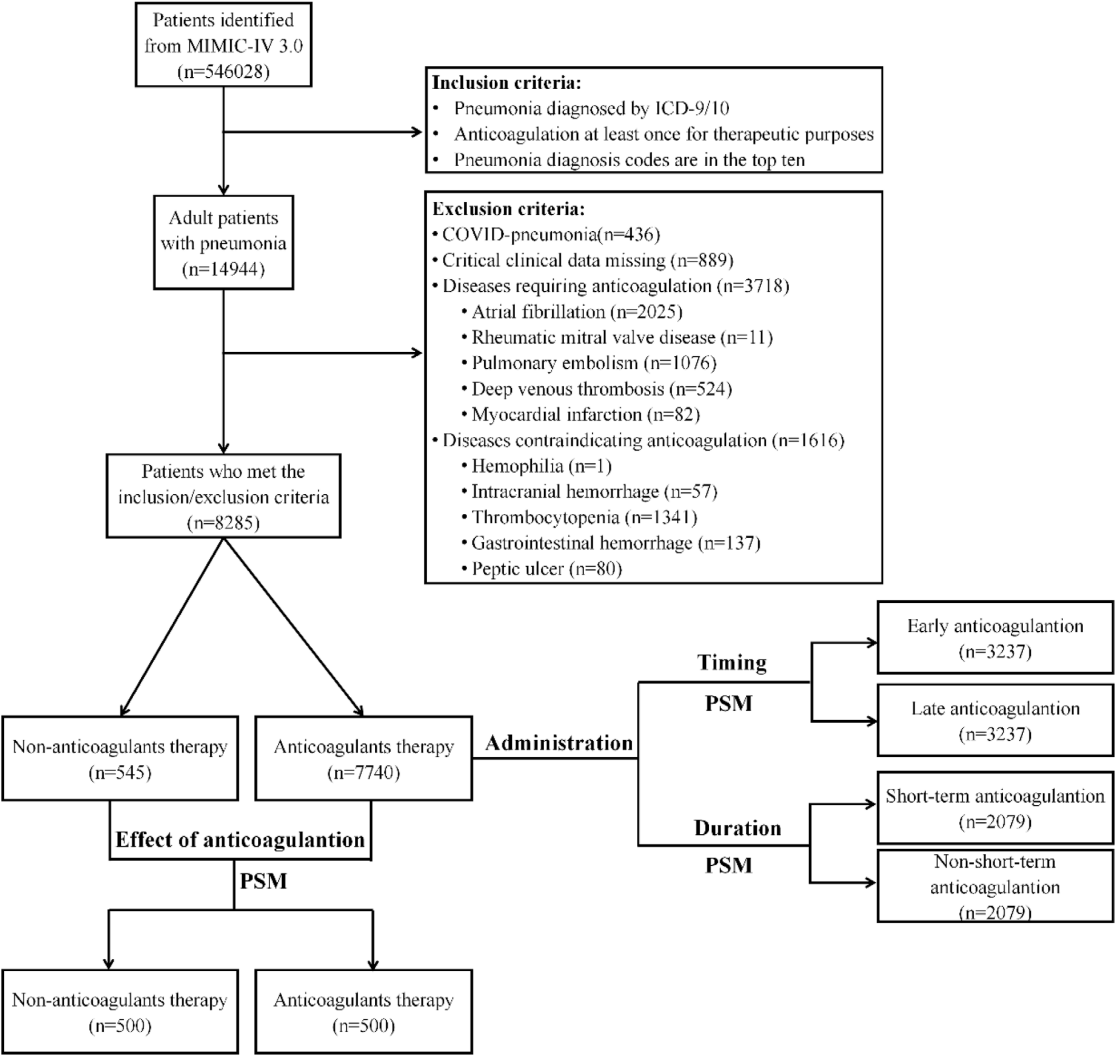
The flowchart of patients included in the analysis.

In this cohort study, we examined adult patients admitted to the intensive care unit (ICU) with a diagnosis of pneumonia, as identified through ICD-9/10 codes. To ensure the relevance of the cases, we included only patients whose ICD diagnostic codes for pneumonia were listed among the top ten diagnoses. The exclusion criteria were as follows: (1) age <18 years; (2) COVID-19 pneumonia patients; (3) critical clinical data missing; (4) apparent errors in clinical data; (5) diseases requiring chronic anticoagulation, such as deep vein thrombosis and atrial fibrillation; (6) diseases contraindicating anticoagulation, such as intracranial hemorrhage and thrombocytopenia. To maintain the integrity of the data, we counted multiple ICU stays for the same patient during a single hospitalization as a single stay. The flowchart is shown in Figure 1.

### 2.3 Data collection and definitions

Data were extracted from MIMIC-Ⅳ using Structured Query Language (SQL) with Navicat Premium (version 13.3.2). The following patient admission data were systematically retrieved: (1) Demographic information: age and sex; (2) Vital signs: heart rate, systolic blood pressure (SBP), diastolic blood pressure (DBP), temperature, respiratory rate (RR) and peripheral oxygen saturation (SpO2); (3) Laboratory data: white blood cell count (WBC), blood platelet count, hemoglobin, creatinine, international normalized ratio (INR), prothrombin time (PT) and partial thromboplastin time (PTT); (4) Clinical scores: the Charlson Comorbidity Index (CCI), simplified acute physiology score (SAPSⅡ score) and sequential organ failure assessment score (SOFA score); (5) Comorbidities: hypertension, asthma, diabetes, heart failure, chronic obstructive pulmonary disease (COPD), liver disease, renal disease, and tumors; (6) Therapeutic interventions: antiplatelet therapy, antipathogen therapy, mechanical ventilation and glucocorticoid use. Additionally, the mortality (7-day mortality and 30-day mortality) were also collected.

The primary outcome for this study was short-term mortality (7-day). Anticoagulant therapy was defined as the use of anticoagulants for clinical intervention during hospitalization. The comorbidities of patients in the MIMIC-Ⅳ database were ascertained based on their recorded diagnoses. Liver diseases included liver cirrhosis, liver failure and other related conditions. Renal diseases included acute kidney injury, uremia, and other related conditions. Tumors encompass a diverse range of malignancies, including but not limited to liver cancer and lung cancer. Antiplatelet therapy included aspirin, tirofiban, clopidogrel, and other related agents. Antipathogen therapy included both antiviral and antibiotic agents, including but not limited to zanamivir, oseltamivir, amoxicillin, clindamycin, levofloxacin, and other relevant antimicrobials. Glucocorticoid therapy included the use of dexamethasone, prednisone, hydrocortisone, and other similar corticosteroids. Anticoagulant therapy included warfarin, heparin, enoxaparin, apixaban, and other related anticoagulants.

### 2.4 Statistical analysis

The study population was categorized into two groups: prophylactic anticoagulant therapy (intervention) and non-anticoagulant therapy (control), according to their treatment status throughout the ICU stay. Continuous variables are presented as mean ± SD for normally distributed data and as median [IQR] for non-normally distributed data. Categorical variables were expressed in numbers and percentages (%). Baseline comparisons between the intervention and control groups were performed using the t-test or Mann-Whitney U test for continuous variables, and the Pearson Chi-Square (χ2) test for categorical variables.

To reduce confounding factors and baseline disparities, PSM was utilized with a caliper width of 0.05 logits of the standard deviation, aligning with methodologies used in prior research (Xu et al., 2024). To preserve data authenticity, individuals with missing variables were excluded. Additionally, certain variables, like D-dimer and C-reactive protein (CRP), were also excluded due to a high proportion of missing values. The cohort was matched in a 1:1 ratio using the nearest neighbor matching technique. Univariate Cox regression analysis was performed to identify variable associations with prognosis and to exclude those with statistical insignificance. The remaining variables were then included in stepwise Cox regression analysis to construct the multivariate Cox model, which was used to evaluate short-term mortality in ICU patients with non-COVID-19. The E-value is calculated based on the observed association between anticoagulant therapy and mortality, which represents the minimum strength of association that an unmeasured confounder would need to have with both the exposure and the outcome, given the measured covariates, in order to completely explain away the observed exposure-outcome association (Haneuse et al., 2019; VanderWeele and Ding, 2017).

Kaplan-Meier curves were generated to depict survival rates according to anticoagulant use, with differences evaluated using the log-rank test. The relationship between WBC, platelet, INR, PTT and the risk of short-term mortality was examined using four-knot restricted cubic spline (RCS) curves based on Cox proportional hazards models. To further explore the optimal anticoagulant administration, subgroup analyses were performed. The subgroup analyses were categorized based on several factors: age (≤60 years and >60 years), sex, WBC counts (≤10 × 10^9^/L and >10 × 10^9^/L), platelet count (≤150 × 10^9^/L and >150 × 10^9^/L), INR (≤2.3 and >2.3), PTT (≤34 s and >34 s), SAPS II score (≤40 and >40), CCI (≤4 and >4), SOFA score (≤7 and >7), comorbidities and therapeutic approaches. Subgroup interactions were evaluated using the variance-ratio test. Furthermore, INR, PTT, WBC counts and platelet counts were stratified into subgroups, guided by the findings from the RCS curve analysis and the distinctive features of the data.

All statistical tests were performed using R software (version 4.4.1). Statistical significance was set at a two-tailed p-value <0.05. And the hazard ratios (HR) were reported along with 95% confidence interval (95% CI).

## 3 Results

### 3.1 Patient characteristics

This study enrolled 8,285 individuals diagnosed with non-COVID-19 pneumonia who met the inclusion criteria. Finally, 500 matched pairs of patients were identified. Among the enrolled subjects, 580 (58.0%) were male and 420 (42.0%) were female. The median age of the total participants was 65.5 years, with a IQR [55.5 years–77.4 years]. The most common comorbidities between the two groups were hypertension and hypoxemia. After PSM, all baseline variables were well-balanced. The nuclear density maps before and after PSM also demonstrated good comparability between the matched populations (Supplementary Figure S1). Detailed comparisons of the clinical characteristics before and after PSM is presented in Table 1.

**TABLE 1:** Baseline characteristic of non-COVID-19 pneumonia patients before and after PSM.

Character	Original cohort			Matched cohort		
	Non- anticoagulation	Anticoagulation	P-value	Non-anticoagulation	Anticoagulation	P-value
Patients	N = 545	N = 7740		N = 500	N = 500	
Age (years)	65.9 ± 16.0	65.7 ± 16.4	0.831	67.0 [55.1, 77.6]	67.2 [55.6, 77.3]	0.617
Sex			0.028			0.481
Male [n (%)]	331 (60.7)	4318 (55.8)		296 (59.2)	284 (56.8)	
Female [n (%)]	214 (39.3)	3422 (44.2)		204 (40.8)	216 (43.2)	
Vital sign						
Heart Rate [/min]	97.3 ± 21.1	95.2 ± 21.3	0.031	94.0 [82.0, 111.3]	98.0 [85.8, 111.0]	0.067
SBP [mmHg]	122.4 ± 25.1	126.4 ± 24.9	<0.001	121.5 [103.0, 140.0]	120.0 [107.0, 138.0]	0.781
DBP [mmHg]	68.3 ± 18.9	71.8 ± 18.6	<0.001	67.0 [58.0, 79.0]	68.0 [58.8, 80.3]	0.259
RR [/min]	22.2 ± 6.7	22.4 ± 6.8	0.537	21.0 [17.0, 26.0]	22.0 [17.9, 27.0]	0.117
Temperature [℃]	36.8 ± 1.0	36.9 ± 0.9	<0.001	36.8 [36.4, 37.2]	36.8 [36.5, 37.3]	0.186
SpO2 [%]	96.6 ± 4.1	96.3 ± 4.6	0.168	98.0 [95.0, 100.0]	97.0 [94.0, 100.0]	0.131
Laboratory data						
WBC [× 10^9/L]	12.5 ± 17.1	12.8 ± 9.5	0.503	9.9 [5.5, 14.6]	9.8 [6.1, 14.2]	0.681
Platelet [× 10^9/L]	167.2 ± 133.7	237.2 ± 121.1	<0.001	158.0 [54.0, 258.0]	168.0 [98.8, 243.0]	0.058
Hemoglobin [g/L]	9.7 ± 2.4	10.8 ± 2.3	<0.001	9.5 [8.1, 11.5]	9.5 [8.0, 11.3]	0.827
Creatinine [mg/dL]	1.6 ± 1.8	1.5 ± 1.7	0.512	1.0 [0.7, 1.6]	1.1 [0.8, 1.8]	0.103
INR	1.6 ± 1.0	1.4 ± 0.8	<0.001	1.3 [1.1, 1.6]	1.3 [1.2, 1.7]	0.131
PT [sec]	17.6 ± 12.2	15.8 ± 9.1	<0.001	14.3 [12.7, 17.4]	14.5 [12.7, 18.1]	0.409
PTT [sec]	35.0 ± 17.4	35.9 ± 20.6	0.354	30.9 [27.1, 35.5]	31.1 [27.4, 36.5]	0.306
Clinically scores						
CCI	7 ± 3	6 ± 3	0.006	6 [5, 9]	7 [5, 9]	0.499
SAPS II score	43 ± 16	40 ± 14	<0.001	40 [31, 52]	43 [33, 54]	0.225
SOFA score	8 ± 5	6 ± 4	<0.001	7 [4, 10]	7 [4, 10]	0.614
Comorbidity						
Hypertension [n (%)]	241(44.2)	3319(42.9)	0.572	218(43.6)	201(40.2)	0.305
Asthma [n (%)]	48(8.8)	761(9.8)	0.481	46(9.2)	48(9.6)	0.914
COPD [n (%)]	63(11.6)	1114(14.4)	0.077	59(11.8)	68(13.6)	0.447
Hypoxemia [n (%)]	169(31.0)	2638(34.1)	0.156	154(30.8)	174(34.8)	0.201
Diabetes [n (%)]	156(28.6)	2680(34.6)	0.005	145(29.0)	146(29.2)	1.000
Heart failure [n (%)]	157(28.8)	2713(35.1)	0.004	142(28.4)	158(31.6)	0.301
Stroke [n (%)]	3(0.6)	14(0.2)	0.176	3(0.6)	0(0.0)	0.248
Liver disease [n (%)]	5(2.8)	122(1.6)	0.056	13(2.6)	9(1.8)	0.518
Renal disease [n (%)]	33(6.1)	455(5.9)	0.940	30(6.0)	40(8.0)	0.265
Tumor [n (%)]	13(2.4)	67(0.9)	0.001	10(2.0)	8(1.6)	0.812
Treatment						
Antiplatelet therapy [n (%)]	128(23.5)	3475(44.9)	<0.001	128(25.6)	135(27.0)	0.666
Antipathogen therapy [n (%)]	514(94.3)	7672(99.1)	<0.001	481(96.2)	478(95.6)	0.750
Glucocorticoid use [n (%)]	203(37.2)	2943(38.0)	0.753	184(36.8)	213(42.6)	0.070
Mechanical ventilation [n (%)]	258(47.3)	4322(55.8)	<0.001	242(48.4)	245(49.0)	0.899

Continuous variables are presented as median [IQR] for non-normally distributed data and as mean ± SD for normally distributed data. Categorical variables were expressed as the numbers and percentages (%). SBP: systolic blood pressure; DBP: diastolic blood pressure; RR: respiratory rate; SpO2: percutaneous oxygen saturation; WBC: white blood cell; INR: international normalized ratio; PT: Prothrombin Time; PTT: Partial Thromboplastin Time; CCI: Charlson Comorbidity Index; SAPS II score: simplified acute physiology score; SOFA score: sequential organ failure assessment score; COPD: chronic obstructive pulmonary disease.

### 3.2 Effect of anticoagulant therapy on mortality

**FIGURE 2:**
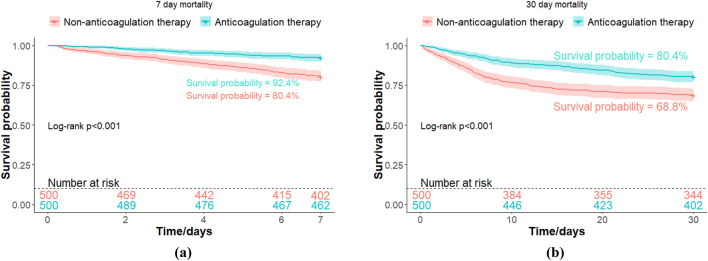
Kaplan-Meier survival curves for mortality of ICU Patients with Non-COVID-19 Pneumonia. **(a)** 7-day mortality; **(b)** 30-day mortality.

Both 7-day mortality (7.6% vs. 19.6%; p < 0.001) and 30-day mortality (19.6% vs. 31.2%; p < 0.001) in the anticoagulant group were lower than non-users. Kaplan-Meier survival analyses also revealed significant improvement in both 7-day and 30-day survival in the anticoagulant group (Figure 2).

### 3.3 Univariate and multivariate cox regression analyses of factors influencing short-term mortality

Univariate analysis suggested that fourteen potential variables independently influence the prognosis of ICU patients with non-COVID-19 pneumonia. Variables with statistical significance in the univariate analysis or clinically significant were included in the stepwise Cox regression. Finally, there were eleven variables were included in the multivariate Cox regression. The eleven variables are age, sex, WBC count, platelet, PTT, SAPS II and SOFA scores, hypoxemia, mechanical ventilation, anticoagulant and antiplatelet therapies (Table 2). The multivariate analysis revealed that male (HR, 0.70; 95% CI, 0.49-1.00; p = 0.048), anticoagulant (HR, 0.30; 95% CI, 0.21-0.44; p < 0.001) and antiplatelet therapies (HR, 0.50; 95% CI, 0.31-0.79; p = 0.003) were both associated with a significantly reduced risk of short-term mortality. Conversely, advanced age (HR, 1.03; 95% CI, 1.02-1.04; p < 0.001), elevated PTT (HR, 1.01; 95% CI, 1.00-1.02; p = 0.004), higher SAPS II scores (HR, 1.04; 95% CI, 1.02-1.05; p < 0.001) and hypoxemia (HR, 1.76; 95% CI, 1.24-2.50; p = 0.001) were all linked to an increased rate of 7-day mortality (Table 2). The E-value for anticoagulant therapy improving short-term mortality of ICU patients with non-COVID-19 pneumonia was 3.97, as was the E-value for the 95%CI (2.91). Notably, creatinine, INR, PT, and CCI were significant in univariate Cox regression analysis but were excluded during stepwise regression. This suggests the presence of multicollinearity between these variables and other covariates. Supplementary Figure S2 displays the outcomes of the multivariate Cox regression analysis along with the corresponding forest plot.

**TABLE 2:** Univariate and multivariate analysis with Cox regression on short-term mortality.

Short-term mortality		Univariate analysis		p-value	Multivariate analysis	p-value
		No.	HR (95%CI)		HR (95%CI)	
Age		1000	1.03 (1.01,1.04)	<0.001	1.03 (1.02,1.04)	<0.001
Sex	Male	580	0.61 (0.43,0.85)	0.004	0.70 (0.49,1.00)	0.048
	Female	420				
WBC counts		1000	1.01 (1.00,1.02)	0.013	1.01 (1.00,1.01)	0.065
Platelet		1000	0.99 (0.99,1.00)	0.264	1.00 (1.00,1.00)	0.092
Creatinine		1000	1.08 (1.00,1.15)	0.039		
INR		1000	1.17 (1.07,1.27)	<0.001		
PT		1000	1.01 (1.00,1.02)	0.004		
PTT		1000	1.01 (1.00,1.02)	0.002	1.01 (1.00,1.02)	0.004
CCI		1000	1.06 (1.00,1.12)	0.047		
Saps II score		1000	1.05 (1.04,1.06)	<0.001	1.04 (1.02,1.05)	<0.001
SOFA score		1000	1.15 (1.12,1.19)	<0.001	1.05 (0.99,1.12)	0.119
Asthma	Yes	94	0.50 (0.24,1.08)	0.077		
	No	906				
Hypoxemia	Yes	328	2.27 (1.58,3.24)	<0.001	1.76 (1.24,2.50)	0.001
	No	672				
Anticoagulant therapy	Yes	500	0.36 (0.25,0.53)	<0.001	0.30 (0.21,0.44)	<0.001
	No	500				
Antiplatelet therapy	Yes	263	0.57 (0.37,0.89)	0.013	0.50 (0.31,0.79)	0.003
	No	737				
Antipathogen therapy	Yes	959	1.10 (0.45,2.68)	0.841		
	No	41				
Glucocorticoid therapy	Yes	397	1.09 (0.78,1.53)	0.617		
	No	603				
Mechanical ventilation	Yes	487	2.27 (1.58,3.24)	<0.001	1.44 (0.93,2.22)	0.101
	No	513				

### 3.4 Subgroup analysis of the impact of anticoagulant therapy on short-term mortality

**FIGURE 3:**
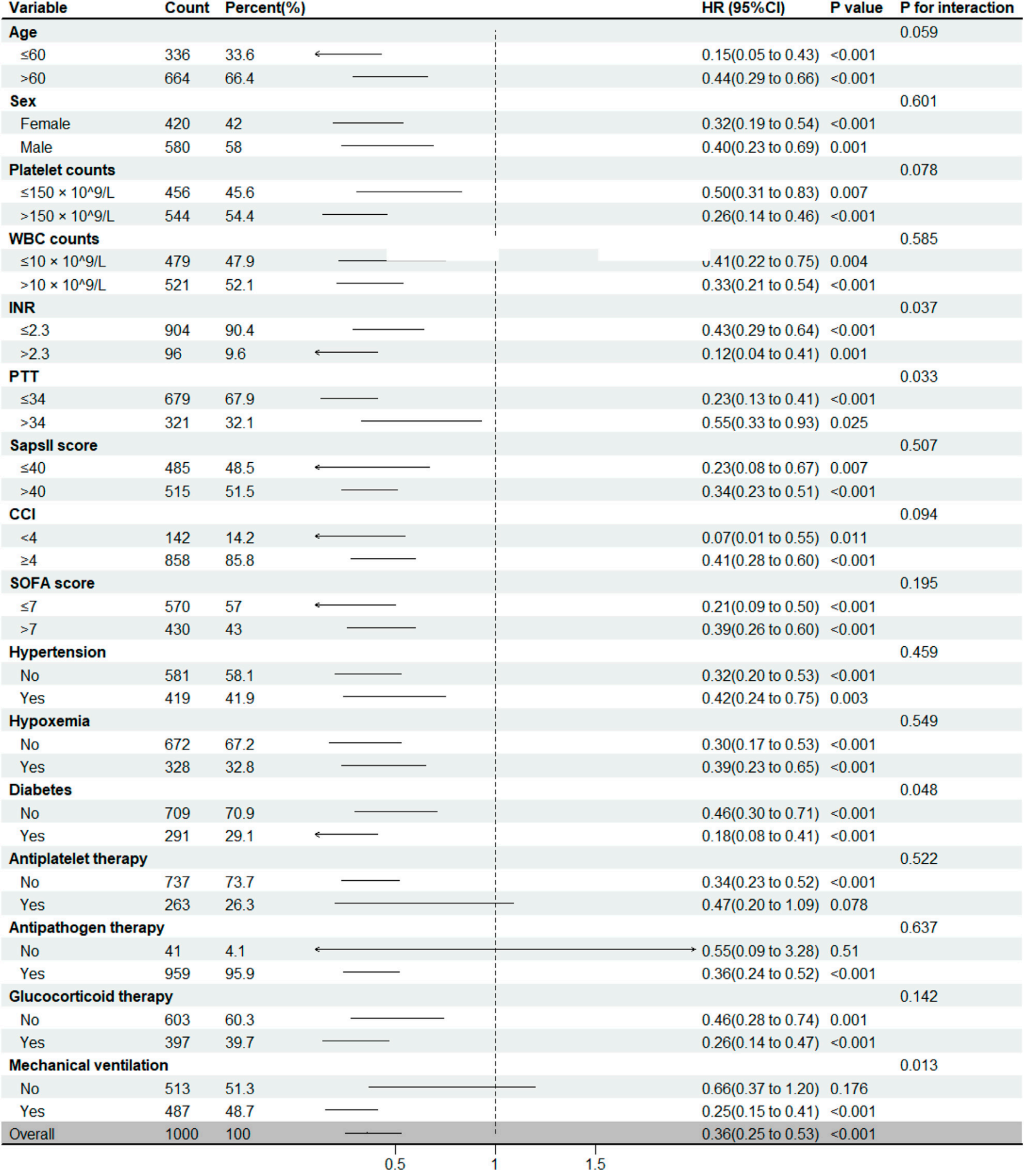
Subgroup analysis of the association between anticoagulation therapy and short-term mortality in ICU Patients with Non-COVID-19 Pneumonia. HR, hazard ratio; WBC, white blood cell; INR, international normalized ratio; PTT, Partial Thromboplastin Time; SAPS II score, simplified acute physiology score II; CCI, Charlson Comorbidity Index; SOFA score, sequential organ failure assessment score.

To further investigate the relationship between anticoagulant therapy and short-term mortality in ICU patients with non-COVID-19 pneumonia, we conducted subgroup analyses informed by the RCS curves (Supplementary Figure S3) and distinctive data characteristics. The results consistently demonstrated that anticoagulant therapy was associated with reduced short-term mortality across most subgroups. Among ICU patients with non-COVID-19 pneumonia, we found that prophylactic anticoagulant therapy was more effective in patients with INR >2.3 (p for interaction = 0.037), PTT ≤34 s (p for interaction = 0.033) and in those with diabetes (p for interaction = 0.048) or who received mechanical ventilation (p for interaction = 0.013). No significant differences in treatment effects were observed in other subgroups (Figure 3).

### 3.5 The optimal timing and duration of prophylactic anticoagulant therapy

To investigate the optimal timing and duration of prophylactic anticoagulant therapy in ICU patients with non-COVID-19 pneumonia, the original 7,740 patients receiving anticoagulant therapy were categorized into two groups: timing (early: <6 h vs. ≥ 6 h) and duration (short-term: <7 days vs. ≥ 7 days). After PSM, there were 3,237 and 2,079 pairs for timing and duration groups, respectively. Both the timing and duration groups demonstrated well-balanced baseline variables. Detailed comparisons of the clinical characteristics before and after PSM is presented in Supplementary Table S1 for timing groups and Supplementary Table S2 for duration groups. For timing of anticoagulant therapy, short-term mortality (4.4% vs. 6.3%; p = 0.001) in the late anticoagulant group was lower than early anticoagulant group, but there was no significant difference for 30-day mortality (15.2% vs. 16.5%; p = 0.163) (Supplementary Table S3). Kaplan-Meier survival analyses also revealed significant improvement in 7-day survival in the late anticoagulant group (Supplementary Figure S4). We further performed univariate COX analyses for 7-day mortality (HR, 0.69; 95% CI, 0.56-0.86; p < 0.001) and 30-day mortality (HR, 0.91; 95% CI, 0.80-1.02; p = 0.100), and the results indicated that late anticoagulant therapy showed a stronger protective effect than early anticoagulant therapy in short-term mortality (Supplementary Table S1). For duration of anticoagulant therapy, the 30-day mortality in the non-short-term anticoagulant group were lower than short-term anticoagulant group (10.2% vs. 23.7%; p < 0.001) (Supplementary Table S2). Kaplan-Meier survival analyses also revealed significant improvement in 30-day survival in the non-short-term anticoagulant group (Supplementary Figure S4). Univariate COX analysis for 30-day mortality indicated that non-short-term anticoagulant therapy was associated with a significantly reduced risk of 30-day mortality (HR, 0.38; 95% CI, 0.32-0.44; p < 0.001) (Supplementary Table S3).

## 4 Discussion

Anticoagulants have been found to play roles beyond their ability to interfere with coagulation processes including anti-inflammatory and antibacterial roles (Wang et al., 2022; Sharma et al., 2014). Pathogen infection triggers an inflammatory response through the sensing of PAMPs and DAMPs by pattern recognition receptors such as toll-like receptors and NOD-like receptors. The recognition translates into an inflammatory response that aims to eliminate the invading pathogen. However, this inflammatory response also interacts with the coagulation system, potentially exacerbating each other and resulting in a state of uncontrolled inflammation and coagulation disorders (Paar et al., 2021). COVID-19 pneumonia is characterized by a dysregulated immune-inflammatory response, marked by cytokine storm, coupled with endothelial dysfunction and hypercoagulability, and increased venous thromboembolic complications (Cron et al., 2021). Current studies have suggested the beneficial effect of correcting coagulation disorders and inflammatory disorders in patients with infection (Pirracchio et al., 2024; Shute, 2023), particularly in COVID-19 and critically ill patients such as ARDS (Cornet et al., 2013; Goligher et al., 2023; Donato, 2021). In non-COVID-19 pneumonia, the interaction between inflammation and coagulation also remains pathophysiologically significant. The infection-driven inflammatory response can activate coagulation pathways, particularly in severe pneumonia. Prophylactic anticoagulation may modulate this inflammatory-coagulative interplay, prevent excessive thrombus formation, and thus reduce vascular complications and improve outcomes. An observational study involving 9,075 patients revealed that the administration of antithrombin can reduce in-hospital mortality rates in severe pneumonia patients complicated by DIC (40.6% vs. 44.2%; p = 0.02) (Tagami et al., 2014). However, current research on anticoagulant therapy primarily concentrates on COVID-19, with relatively less focus on non-COVID-19 pneumonia.

In this retrospective study leveraging the MIMIC-Ⅳ database, we explored the potential impact of prophylactic anticoagulant therapy in ICU patients with non-COVID-19 pneumonia. Kaplan-Meier survival analyses also revealed improvement in both 7-day and 30-day survival in patients receiving prophylactic anticoagulant treatment. Both univariate and multivariate Cox regression analyses consistently demonstrated a reduction in short-term mortality associated with prophylactic anticoagulant therapy. The E-value of this association was 3.97, indicating that it is unlikely that an unmeasured variable explained the observed association between anticoagulant therapy and mortality. Notably, antiplatelet therapy (HR, 0.50; 95% CI, 0.31-0.79; p = 0.003) also improved the mortality, similar to the conclusion of a recently published retrospective study (HR, 0.75; 95% CI, 0.63-0.88; p < 0.001) (Wang et al., 2024). In multivariate analysis, advanced age, elevated PTT, higher SAPS II scores and hypoxemia were all linked to an increased rate of short-term mortality. These findings imply that dysfunctional coagulation and severe disease status are key factors linked to a poor prognosis.

To explore the characteristics and heterogeneity of anticoagulants in different populations, RCS curve and subgroup analyses were conducted. Among ICU patients with non-COVID-19 pneumonia, prophylactic anticoagulant therapy was associated with reduced short-term mortality across most subgroups. We found that prophylactic anticoagulant therapy was more effective in patients with INR >2.3, PTT ≤34 s, as well as in those with diabetes or requiring mechanical ventilation. These results suggest that anticoagulant therapy might be more advantageous for individuals whose coagulation function is within a relatively normal range. Interestingly, anticoagulant therapy also showed greater efficacy in the diabetes subgroup. In the terminal stages of diabetes, autonomic neuropathy may impair the “inflammatory reflex” mechanism, thereby intensifying the inflammatory state and further exacerbating vascular damage (Tracey, 2002; Fuso et al., 2019). This enhanced efficacy be attributed to the routine use of metformin in diabetic patients, which likely helps reduce the incidence of cardiovascular complications to some extent (Mach et al., 2020). To identify the optimal drug administration regimen, we also compared both the timing and duration of anticoagulants treatment. Kaplan-Meier survival analyses revealed significant improvements in survival in both the late and non-short-term anticoagulant groups, aligning with the results of univariate Cox analyses. These findings suggest that the benefits of prophylactic anticoagulant therapy might be amplified in ICU patients with non-COVID-19 pneumonia, especially when patients experiencing combined infection and inflammation responses.

Notably, this study has some limitations that warrant consideration. First, our samples and the primary diagnostic criteria are derived from a single database. The absence of external validation with additional data limits the broader applicability of our findings. Secondly, due to significant data unavailability, certain laboratory variables—such as CRP, interleukin-6 (IL-6), and D-dimer—were not included in our analyses. These markers are crucial for a comprehensive understanding of the clinical status of patients. However, we carefully controlled for known confounding factors through PSM and our sensitivity analyses showed an E-value of 3.97 for the effect of anticoagulant therapy on short-term mortality in ICU patients with non-COVID-19 pneumonia. This high E-value suggests that our findings are robust and unlikely to be explained by residual confounding alone. Finally, the characteristics of the database, such as a large number of patients with unknown pathogens in the diagnostic codes and the features of a retrospective study, precluded us from conducting further subgroup analyses.

## 5 Conclusion

This retrospective study indicates that prophylactic anticoagulant therapy is associated with a reduction in mortality among ICU patients with non-COVID-19 pneumonia. Despite the potential beneficial effects of prophylactic anticoagulant therapy, future research, randomized controlled clinical trials (RCTs), are essential to provide a more comprehensive understanding of the impact of anticoagulant therapy.

## Data Availability

Publicly available datasets were analyzed in this study. This data can be found here: The original contributions presented in MIMIC database. Please note that access to the MIMIC database requires completing relevant training and obtaining proper permission. Detailed information on accessing the MIMIC-IV database can be found at https://mimic.physionet.org/; DOI: 10.13026/6mm1-ek67. Further inquiries can be directed to Dr Hu.

## Abbreviations

COVID-19, Coronavirus disease 2019; MIMIC-Ⅳ, Medical Information Mart for Intensive Care Ⅳ; SBP, systolic blood pressure; DBP, diastolic blood pressure; RR, respiratory rate; SpO2, percutaneous oxygen saturation; WBC, white blood cell; INR, international normalized ratio; PT, Prothrombin Time; PTT, Partial Thromboplastin Time; CCI, the Charlson Comorbidity Index; SAPSⅡ score, simplified acute physiology score; SOFA score, sequential organ failure assessment score; COPD, chronic obstructive pulmonary disease; RCS, restricted cubic spline.
