# Supplementary material for: Prophylactic anticoagulant therapy is associated with improved survival in ICU patients with non-COVID-19 pneumonia: a retrospective cohort study
Source: Front Pharmacol. 2025 May 8;16:1597885. doi: 10.3389/fphar.2025.1597885 (PMC12095157; doi:10.3389/fphar.2025.1597885)
Supplement: Supplementary file 1 [file DataSheet1.docx]

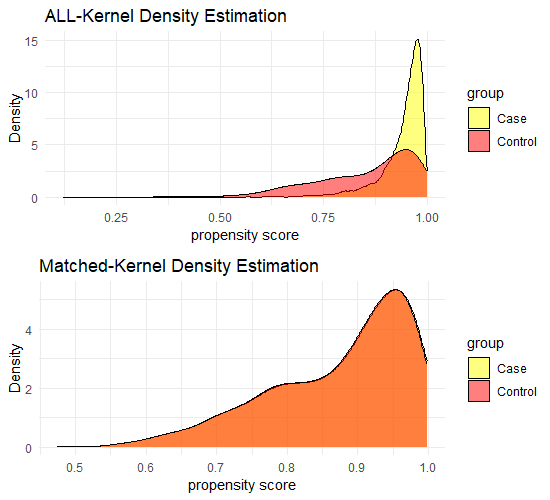


**Figure S1** The nuclear density maps before and after PSM matching. PSM: propensity score matching


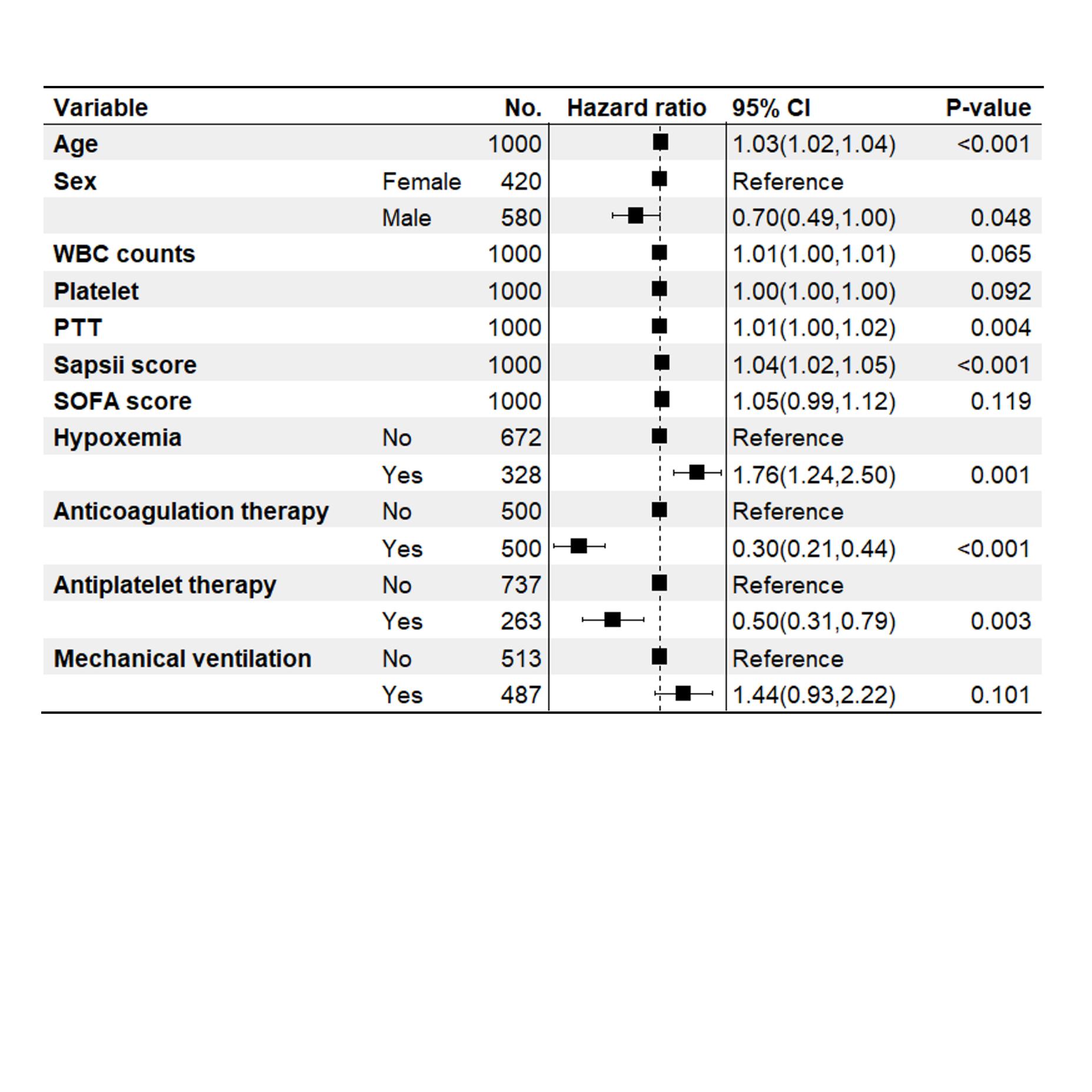


**Figure S2** Multivariate analysis with Cox regression for short-term mortality and corresponding forest plot. INR: international normalized ratio; PTT: Partial Thromboplastin Time; SAPS ii score: simplified acute physiology score


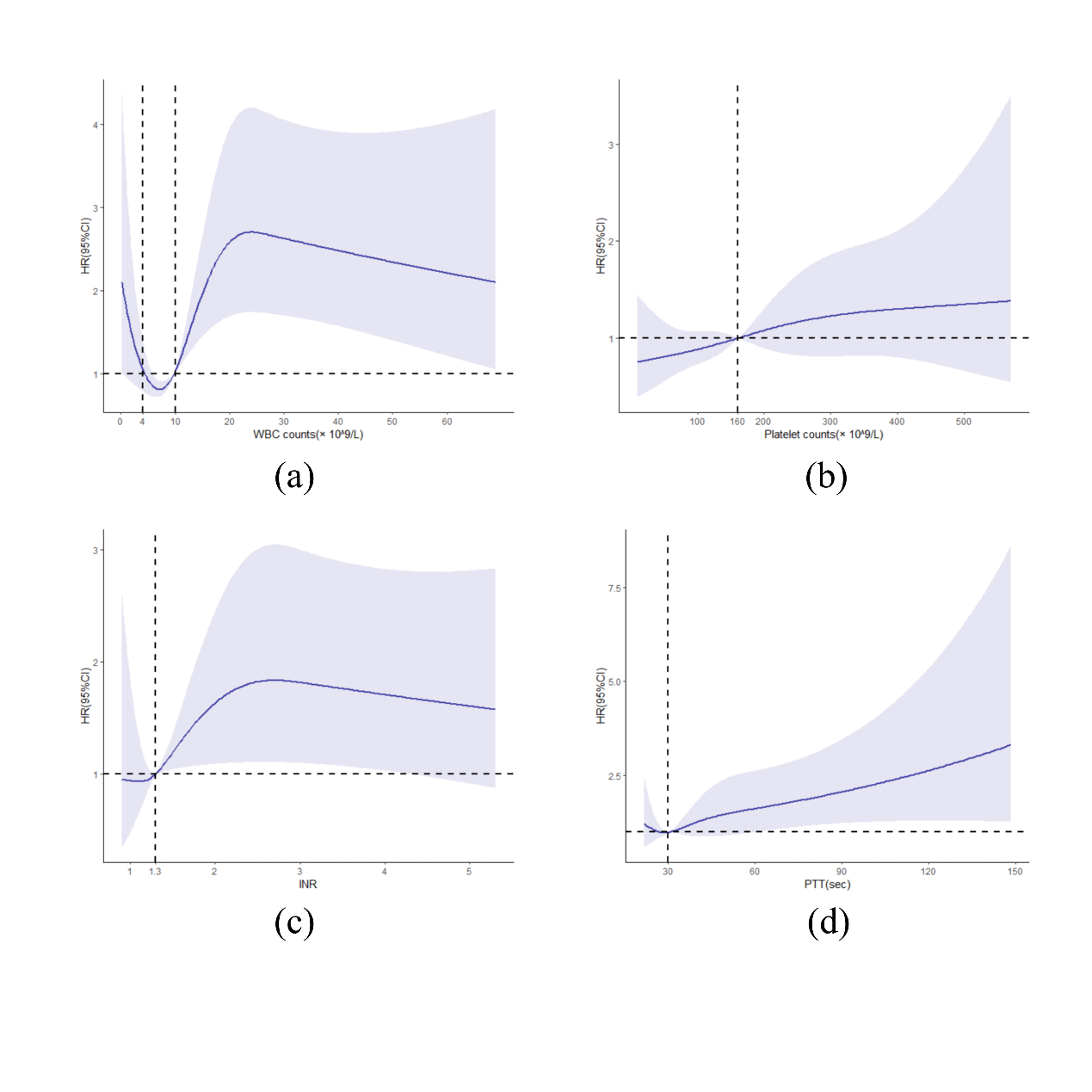


**Figure S3** Restricted cubic spline (RCS) curves analysis of laboratory parameters-related indicators and short-term mortality. (a) WBC count; (b) Platelet count; (c) INR; (d) PTT


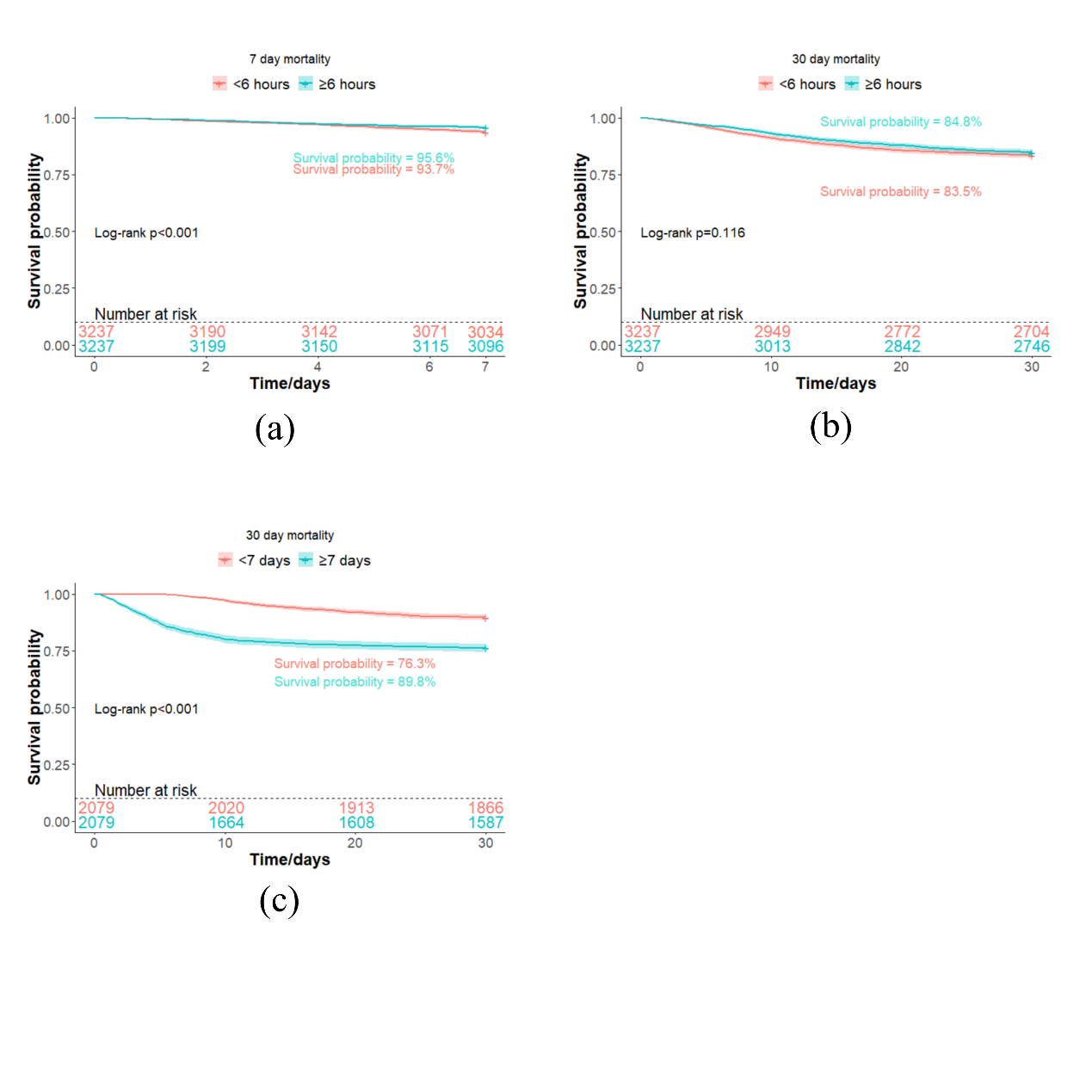


**Figure S4** Kaplan-Meier survival curves for mortality of ICU Patients with Non-COVID-19 Pneumonia. (a) Timing (early vs late) and 7-day mortality; (b) Timing (early vs late) and 30-day mortality; (c) Duration (short-term vs long-term) and 30-day mortality

**Table S1** The demographic and clinical characteristics of non-COVID-19 pneumonia patients before and after PSM according to timing of anticoagulation therapy.

| **Character** | **Original cohort** | | **P-value** | **Matched cohort** | | **P-value** |
| --- | --- | --- | --- | --- | --- | --- |
|  | **Early** | **Late** |  | **Early** | **Late** |  |
| **Patients** | N=3830 | N=3910 |  | N=3237 | N=3237 |  |
| Age (years) | 65.9±16.4 | 65.6±16.5 | 0.310 | 65.8±16.5 | 65.6±16.8 | 0.789 |
| Sex |  |  | 0.024 |  |  | 0.841 |
| Male [n (%)] | 2087 (54.5) | 2231 (57.1) |  | 1800 (55.6) | 1809 (55.9) |  |
| Female [n (%)] | 1743 (45.5) | 5409 (42.9) |  | 1437 (44.4) | 1428 (44.1) |  |
| **Vital sign** |  |  |  |  |  |  |
| Heart Rate [/min] | 95.5±21.1 | 95.0±21.5 | 0.268 | 95.3±21.1 | 95.6±21.4 | 0.573 |
| SBP [mmHg] | 125.0±24.7 | 127.8±25.1 | <0.001 | 126.0±24.9 | 126.1±24.5 | 0.822 |
| DBP [mmHg] | 71.6±18.9 | 72.0±18.4 | 0.414 | 72.1±19.1 | 71.5±18.3 | 0.140 |
| RR [/min] | 22.7±6.7 | 22.1±6.9 | <0.001 | 22.4±6.5 | 22.5±7.1 | 0.611 |
| Temperature [℃] | 37.0±0.8 | 36.9±0.9 | 0.031 | 36.9±0.9 | 37.0±0.8 | 0.918 |
| SpO2 [%] | 96.0±4.94 | 96.7±4.2 | <0.001 | 96.4±4.1 | 96.4±4.2 | 0.804 |
| **Laboratory data** |  |  |  |  |  |  |
| WBC [× 10^9/L] | 12.8±8.8 | 12.8±10.1 | 0.980 | 12.6±8.3 | 12.8±10.2 | 0.498 |
| Platelet [× 10^9/L] | 242.3±121.9 | 232.2±120.0 | <0.001 | 237.7±114.4 | 238.3±121.9 | 0.818 |
| Hemoglobin [g/L] | 10.8±2.3 | 10.8±2.3 | 0.943 | 10.8±2.3 | 10.8±2.3 | 0.913 |
| Creatinine [mg/dL] | 1.6±1.8 | 1.5±1.7 | 0.020 | 1.6±1.7 | 1.6±1.8 | 0.981 |
| INR | 1.4±0.7 | 1.5±1.0 | <0.001 | 1.4±0.7 | 1.4±0.8 | 0.164 |
| PT [sec] | 15.2±7.1 | 16.4±10.7 | <0.001 | 15.4±7.6 | 15.7±8.1 | 0.138 |
| PTT [sec] | 37.0±21.9 | 34.8±19.1 | <0.001 | 35.3±18.1 | 35.1±19.8 | 0.686 |
| **Clinically scores** |  |  |  |  |  |  |
| CCI | 6±3 | 6±3 | 0.892 | 6.3±3.1 | 6.3±3.1 | 0.846 |
| SAPS II score | 40±14 | 40±15 | 0.896 | 40.5±14.5 | 40.0±14.5 | 0.168 |
| SOFA score | 6±4 | 6±4 | 0.919 | 6.5±4.0 | 6.4±3.9 | 0.075 |
| **Comorbidity** |  |  |  |  |  |  |
| Hypertension [n (%)] | 1562(40.8) | 1757(44.9) | <0.001 | 1366(42.2) | 1378(42.6) | 0.782 |
| Asthma [n (%)] | 415(10.8) | 346(8.8) | 0.004 | 335(10.3) | 317(9.8) | 0.483 |
| COPD [n (%)] | 610(15.9) | 504(12.9) | <0.001 | 458(14.1) | 460(14.2) | 0.972 |
| Hypoxemia [n (%)] | 1413(36.9) | 1225(31.3) | <0.001 | 1110(34.3) | 1112(34.4) | 0.979 |
| Diabetes [n (%)] | 1392(36.3) | 1288(32.9) | 0.002 | 1126(34.8) | 1123(34.7) | 0.958 |
| Heart failure [n (%)] | 1489(38.9) | 1224(31.3) | <0.001 | 1118(34.5) | 1130(34.9) | 0.774 |
| Stroke [n (%)] | 6(0.2) | 8(0.2) | 0.819 | 5(0.2) | 4(0.1) | 1.000 |
| Liver disease [n (%)] | 56(1.5) | 66(1.7) | 0.480 | 47(1.5) | 53(1.6) | 0.614 |
| Renal disease [n (%)] | 241(6.3) | 214(5.5) | 0.138 | 188(5.8) | 185(5.7) | 0.915 |
| Tumor [n (%)] | 35(0.9) | 32(0.8) | 0.741 | 31(1.0) | 29(0.9) | 0.897 |
| **Treatment** |  |  |  |  |  |  |
| Antiplatelet therapy [n (%)] | 1822(47.6) | 1653(42.3) | <0.001 | 1432(44.2) | 1438(44.4) | 0.900 |
| Antipathogen therapy [n (%)] | 3791(99.0) | 3881(99.3) | 0.237 | 3206(99.0) | 3211(99.2) | 0.595 |
| Glucocorticoid use [n (%)] | 1500(39.2) | 1443(36.9) | 0.043 | 1237(38.2) | 1236(38.2) | 1.000 |
| Mechanical ventilation [n (%)] | 2017(52.7) | 2305(59.0) | <0.001 | 1786(55.2) | 1799(55.6) | 0.764 |
| **Clinical Outcomes** |  |  |  |  |  |  |
| 7-day mortality | 236(6.2) | 171(4.4) | 0.001 | 203(6.3) | 141(4.4) | 0.001 |
| 30-day mortality | 619(16.2) | 596(15.2) | 0.280 | 533(16.5) | 491(15.2) | 0.163 |

Continuous variables are presented as mean ± SD for normally distributed data. Categorical variables were expressed as the numbers and percentages (%). SBP: systolic blood pressure; DBP: diastolic blood pressure; RR: respiratory rate; SpO2: percutaneous oxygen saturation; WBC: white blood cell; INR: international normalized ratio; PT: Prothrombin Time; PTT: Partial Thromboplastin Time; CCI: Charlson Comorbidity Index; SAPS II score: simplified acute physiology score; SOFA score: sequential organ failure assessment score; COPD: chronic obstructive pulmonary disease; MV: Mechanical ventilation; GH: Gastrointestinal hemorrhage; Timing (early: < 6 hours and late: ≥ 6 hours)

**Table S2** The demographic and clinical characteristics of non-COVID-19 pneumonia patients before and after PSM according to duration of anticoagulation therapy.

| **Character** | **Original cohort** | | **P-value** | **Matched cohort** | | **P-value** |
| --- | --- | --- | --- | --- | --- | --- |
|  | **Short-term** | **Non-short-term** |  | **Short-term** | **Non-short-term** |  |
| **Patients** | N=2098 | N=5640 |  | N=2079 | N=2079 |  |
| Age (years) | 65.9±16.0 | 65.7±16.4 | 0.831 | 67.0±17.1 | 67.1±15.9 | 0.951 |
| Sex |  |  | 0.001 |  |  | 0.877 |
| Male [n (%)] | 1105 (52.7) | 3212 (57.0) |  | 982 (47.2) | 976 (46.9) |  |
| Female [n (%)] | 993 (47.3) | 2428 (43.0) |  | 1097 (52.8) | 1103 (53.1) |  |
| **Vital sign** |  |  |  |  |  |  |
| Heart Rate [/min] | 95.0±20.6 | 95.3±21.6 | 0.562 | 95.0±20.6 | 95.3±21.1 | 0.564 |
| SBP [mmHg] | 124.3±24.8 | 127.1±24.9 | <0.001 | 124.5±24.9 | 125.2±24.3 | 0.328 |
| DBP [mmHg] | 70.5±18.8 | 72.4±18.5 | <0.001 | 70.3±18.8 | 70.7±17.8 | 0.505 |
| RR [/min] | 22.0±6.6 | 22.5±6.9 | 0.003 | 22.0±6.6 | 22.1±6.6 | 0.731 |
| Temperature [℃] | 36.9±0.9 | 37.0±0.8 | <0.001 | 36.9±0.9 | 36.9±0.8 | 0.605 |
| SpO2 [%] | 95.7±5.4 | 96.5±4.3 | <0.001 | 95.9±4.7 | 95.9±4.4 | 0.811 |
| **Laboratory data** |  |  |  |  |  |  |
| WBC [× 10^9/L] | 12.9±9.6 | 12.7±9.4 | 0.419 | 12.9±9.6 | 12.3±10.5 | 0.068 |
| Platelet [× 10^9/L] | 232.2±126.9 | 239.1±118.8 | 0.027 | 232.5±126.9 | 234.9±115.4 | 0.518 |
| Hemoglobin [g/L] | 10.6±2.2 | 10.8±2.3 | <0.001 | 10.6±2.2 | 10.6±2.2 | 0.541 |
| Creatinine [mg/dL] | 1.6±1.7 | 1.5±1.7 | 0.573 | 1.6±1.7 | 1.5±1.8 | 0.733 |
| INR | 1.5±1.0 | 1.4±0.8 | 0.006 | 1.5±1.0 | 1.5±0.8 | 0.291 |
| PT [sec] | 16.3±10.8 | 15.6±8.4 | 0.003 | 16.3±10.4 | 16.0±9.4 | 0.329 |
| PTT [sec] | 36.2±20.6 | 35.8±20.6 | 0.391 | 36.2±20.5 | 35.6±19.8 | 0.334 |
| **Clinically scores** |  |  |  |  |  |  |
| CCI | 6±3 | 6±3 | 0.111 | 6±3 | 7±3 | 0.141 |
| SAPS II score | 39±15 | 41±14 | <0.001 | 39±15 | 39±13 | 0.887 |
| SOFA score | 6±4 | 7±4 | <0.001 | 6±4 | 6±4 | 0.758 |
| **Comorbidity** |  |  |  |  |  |  |
| Hypertension [n (%)] | 868(41.4) | 2449(43.4) | 0.111 | 861(41.4) | 869(41.8) | 0.826 |
| Asthma [n (%)] | 228(10.9) | 533(9.5) | 0.069 | 225(10.8) | 241(11.6) | 0.461 |
| COPD [n (%)] | 290(13.8) | 824(14.6) | 0.401 | 289(13.9) | 333(16.0) | 0.062 |
| Hypoxemia [n (%)] | 638(30.4) | 1998(35.4) | <0.001 | 631(30.4) | 676(32.5) | 0.142 |
| Diabetes [n (%)] | 678(32.3) | 2002(35.5) | 0.010 | 673(32.4) | 692(33.3) | 0.552 |
| Heart failure [n (%)] | 695(33.1) | 2018(35.8) | 0.032 | 690(33.2) | 738(35.5) | 0.125 |
| Stroke [n (%)] | 4(0.2) | 10(0.2) | 1.000 | 4(0.2) | 5(0.2) | 1.000 |
| Liver disease [n (%)] | 31(1.5) | 91(1.6) | 0.746 | 31(1.5) | 32(1.5) | 1.000 |
| Renal disease [n (%)] | 123(5.9) | 332(5.9) | 1.000 | 121(5.8) | 128(6.2) | 0.695 |
| Tumor [n (%)] | 26(1.2) | 41(0.7) | 0.043 | 25(1.2) | 19(0.9) | 0.449 |
| **Treatment** |  |  |  |  |  |  |
| Antiplatelet therapy [n (%)] | 829 (39.5) | 2645 (46.9) | <0.001 | 826(39.7) | 854(41.1) | 0.393 |
| Antipathogen therapy [n (%)] | 2059 (98.1) | 5611 (99.5) | <0.001 | 2052(98.7) | 2055(98.8) | 0.778 |
| Glucocorticoid use [n (%)] | 723 (34.5) | 2219 (39.3) | <0.001 | 721(34.7) | 757(36.4) | 0.257 |
| Mechanical ventilation [n (%)] | 727 (34.7) | 3593 (63.7) | <0.001 | 726(34.9) | 736(35.4) | 0.770 |
| **Clinical Outcome** |  |  |  |  |  |  |
| 30-day mortality | 500 (23.8) | 713 (12.6) | <0.001 | 492(23.7) | 213(10.2) | <0.001 |

Continuous variables are presented as mean ± SD for normally distributed data. Categorical variables were expressed as the numbers and percentages (%). SBP: systolic blood pressure; DBP: diastolic blood pressure; RR: respiratory rate; SpO2: percutaneous oxygen saturation; WBC: white blood cell; INR: international normalized ratio; PT: Prothrombin Time; PTT: Partial Thromboplastin Time; CCI: Charlson Comorbidity Index; SAPS II score: simplified acute physiology score; SOFA score: sequential organ failure assessment score; COPD: chronic obstructive pulmonary disease; MV: Mechanical ventilation; GH: Gastrointestinal hemorrhage. Duration (short-term: < 7 days and non-short-term ≥ 7 days). (Note: Two patients in the original cohort were removed due to missing data for duration)

**Table S3** Univariate analyses with Cox regression on mortality: Impact of Timing and Duration of anticoagulant therapy (after PSM respectively)

| **Variables** |  | | **7-day mortality** | | **p-value** | **30-day mortality** | **p-value** |
| --- | --- | --- | --- | --- | --- | --- | --- |
|  |  | **No.** | | **HR (95%CI)** |  | **HR (95%CI)** |  |
| Timing | Early | 3237 | | Reference |  | Reference |  |
|  | Late | 3237 | | 0.69 (0.56,0.86) | <0.001 | 0.91 (0.80,1.02) | 0.100 |
| Duration | Short-term | 2079 | | Reference |  | Reference |  |
|  | Non-short-term | 2079 | |  |  | 0.38 (0.32,0.44) | <0.001 |

Timing (early: < 6 hours and late: ≥ 6 hours) and Duration (short-term: < 7 days and non-short-term ≥ 7 days)
